# Wavelet analysis of frequency chaos game signal: a time-frequency signature of the *C. elegans* DNA

**DOI:** 10.1186/s13637-014-0016-z

**Published:** 2014-09-12

**Authors:** Imen Messaoudi, Afef Elloumi Oueslati, Zied Lachiri

**Affiliations:** 1 Ecole Nationale d’Ingénieurs de Tunis, LR Signal, Images et Technologies de l’Information, Université de Tunis El Manar, BP 37, le Belvédère, Tunis, 1002 Tunisia; 2 Département de Génie Physique et Instrumentation, INSAT, Centre Urbain Cedex, BP 676, Tunis, 1080 Tunisia

**Keywords:** C. elegans, Complex Morlet wavelet scalogram, Continuous wavelet transform, Frequency chaos game signal, Local signature

## Abstract

Challenging tasks are encountered in the field of bioinformatics. The choice of the genomic sequence’s mapping technique is one the most fastidious tasks. It shows that a judicious choice would serve in examining periodic patterns distribution that concord with the underlying structure of genomes. Despite that, searching for a coding technique that can highlight all the information contained in the DNA has not yet attracted the attention it deserves. In this paper, we propose a new mapping technique based on the chaos game theory that we call the frequency chaos game signal (FCGS). The particularity of the FCGS coding resides in exploiting the statistical properties of the genomic sequence itself. This may reflect important structural and organizational features of DNA. To prove the usefulness of the FCGS approach in the detection of different local periodic patterns, we use the wavelet analysis because it provides access to information that can be obscured by other time-frequency methods such as the Fourier analysis. Thus, we apply the continuous wavelet transform (CWT) with the complex Morlet wavelet as a mother wavelet function. Scalograms that relate to the organism *Caenorhabditis elegans (C. elegans)* exhibit a multitude of periodic organization of specific DNA sequences.

**Electronic supplementary material:**

The online version of this article (doi:10.1186/s13637-014-0016-z) contains supplementary material, which is available to authorized users.

## 1 Introduction

The fundamental information for a living being resides essentially in its nucleic material—the DNA. This molecule contains all the instructions needed to produce proteins and enzymes for all of the metabolic pathways. Thus, revealing the structural and organizational features in DNA sequences is a very interesting topic. However, the search for relevant information along the genomic sequences is not an easy task. In fact, although several programs have been created which aim at detecting valuable information concerning the DNA, there is much work remaining to be done. In order to better understand the genomic sequence role and structure, several signal processing approaches have been investigated. To be able to apply such techniques, it is imperative to convert DNA characters into numerical sequences. This operation is the so-called coding technique. Thereby, various approaches for DNA character coding have been reported including the binary coding [1],[2], the inter-distance signals [3], coding with the entropy measure [4], the electron-ion interaction pseudo-potential (EIIP) mapping [5], the structural bending trinucleotide coding (PNUC) [2], etc.

The choice of the most appropriate coding technique for a desired analysis represents a basic problem. It turns that coding techniques that are based on physical, chemical and structural DNA characteristics are efficient in terms of revealing specific structures as is the case with EIIP and PNUC coding approaches.

Here, we propose a new mapping technique inspired from the Chaos Game theory to which we associate the name of ‘frequency chaos game signals’ (FCGS). The FCGS approach relies on the frequency value of each sub-pattern assignment, which gives us the opportunity to produce several signals for the same input sequence, depending on the size of the considered sub-patterns. The specificity of our coding consists on exploiting the statistical properties of the genomic sequence itself, which may serve in detecting interesting structures within the DNA sequences.

The efficiency of our method in detecting different biological events is demonstrated through application of the continuous wavelet transform (CWT). The choice of such analysis method (we mean CWT) is justified by the need of a time-frequency approach that provides local frequency information which is not guaranteed by other transforms such as the Fourier transform. In fact, the classical Fourier transform does not contain local information. Thus, it appears that the short-time Fourier transform (STFT) is better suited to predict sites with biological relevance in the genomic signals. Nevertheless, this method requires a good choice of the analysis window’s size that must balance the frequency and temporal resolutions. The short Fourier transform induces interferences and loss of information [6]. With the advent of the wavelet transform (WT), one can get more precise and more adequate analysis especially concerning the location of hotspots in signals with complex nature, which is the case of genomic signals [5],[7]-[10].

In this paper, we investigate the role of the CWT in displaying the frequency-dependent structure of genomic signals by using the complex Morlet wavelet scalogram. The purpose of this analysis consists in revealing spectral features that might be of biological significance in the *Caenorhabditis elegans (C. elegans)* genome. This study is particular since it exposes a new coding technique which is efficient in terms of the DNA characterization.

This paper is divided into five sections: First, we describe the steps required to generate the frequency chaos game signals in section 2. In section 3, we deal with the complex wavelet analysis in which we give an overview on the continuous wavelet transform as well as a brief description of the complex Morlet wavelet. In section 4, we analyze the DNA sequences by the Morlet wavelet, and then we expose and discuss the results in section 5. Finally, in section 6, we conclude this paper.

## 2 Introduction to the frequency chaos game signals

Starting from the pioneer work of Jeffrey in 1990, representing DNA sequences by the chaos game representation (CGR) has drawn a resounding success. In fact, for more than 2 decades, the chaos game representation has been used as a platform for pattern recognition [11],[12], a generalization of Markov transition tables [13], a tool for statistical characterization of genomic sequences [11],[14],[15], as well as a basis for alignment comparisons [16] and establishment of phylogenetic trees [17]. The CGR is an iterative algorithm that provides unique scatter picture of fractal nature. It consists on mapping a nucleotide sequence in a unit-square, where each of its vertices is assigned to a DNA character (nucleotides: A, C, G and T). Let us consider a given DNA sequence composed of *N* nucleotides *S*={ *S*_1_,*S*_2_,….,*S*_*N*_}. Thus, an element occupying the *i* th position in *S* is represented into the square by a point *x*_*i*_. The point *x*_*i*_ is repeatedly placed halfway between the previous plotted point *x*_*i*−1_ and the segment joining the vertex corresponding to the read letter *S*_*i*_[18]. The prolific iterative function of CGR is given by

1 $$\begin{array}{ll} \left\{\begin{array}{c} x_{0}=(0.5,0.5)\\ x_{i}=x_{i-1}+\frac{1}{2}(y_{i}-x_{i-1}),i=1,\ldots ,N \end{array}\right.\;\\ \;\text{where}\;y_{i}= & \left\{\begin{array}{c} (0,0)\;\text{if}\;\;S[i]=A\\ (0,1)\;\text{if}\;\;S[i]=C\\ (1,0)\;\text{if}\;\;S[i]=T\\ (1,1)\;\text{if}\;\;S[i]=G \end{array}\right.\; \end{array}$$

**Figure 1:**
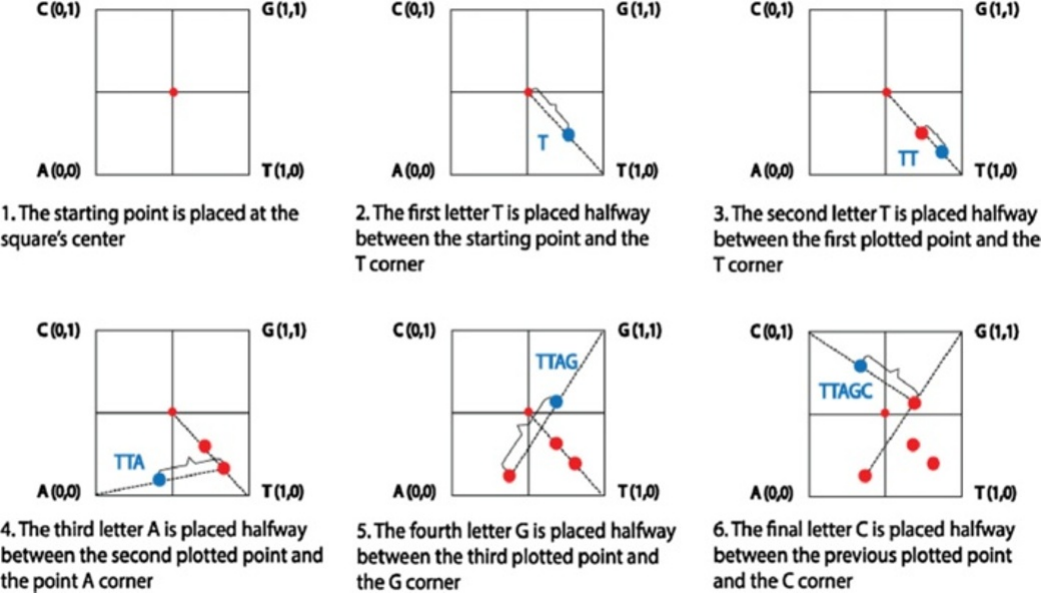
**Illustration of the** ***CGR*** **process to represent the input sequence ‘TTAGC’.**

Usually, the starting point *x*_0_ is placed at the center of the square while the choice of the corners is arbitrary and can be assigned in any other way. The figure given below (Figure 1) shows the procedure to draw the sequence ‘TTAGC’.

The usefulness of the chaos game representation goes beyond the convenience of genome representation and visualization. In addition, it provides a unique image which is specific to the considered genome [19],[20] and thus forms an outstanding genomic signature [21].

**Figure 2:**
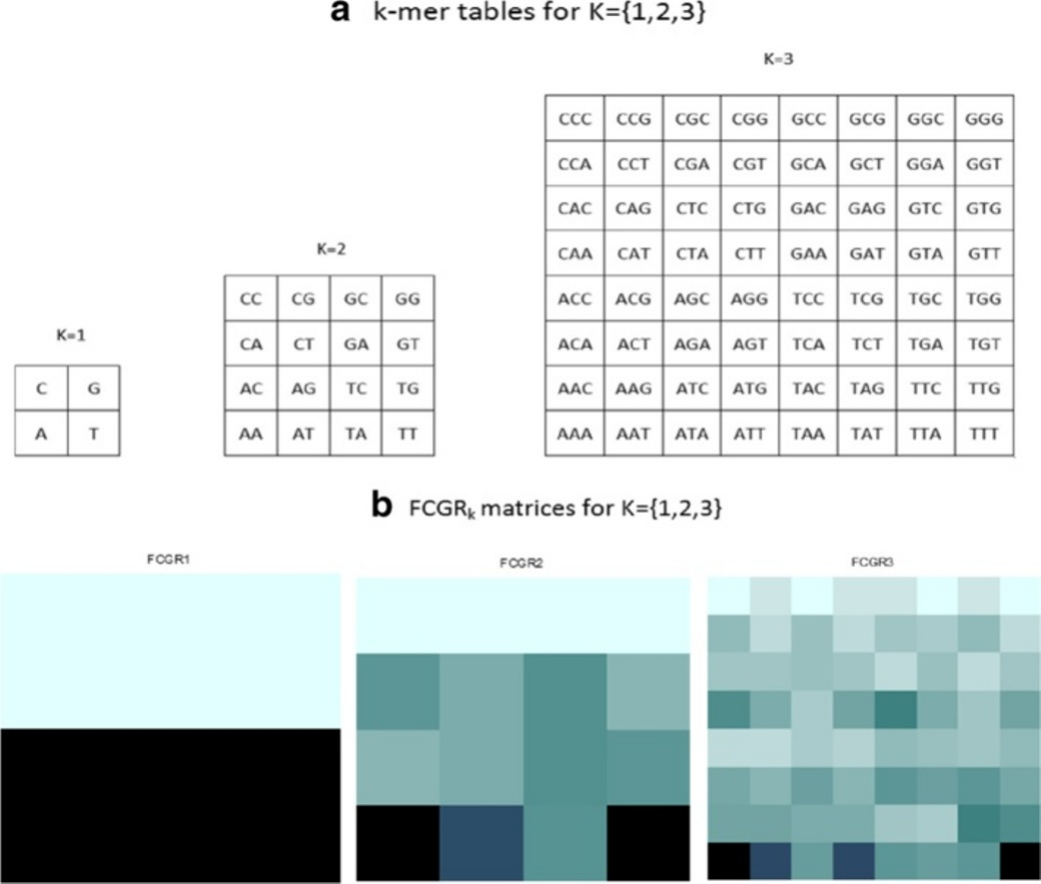
**Definition of the** ***k*** **-mer tables for** ***k={1,2,3}*** **(a) and representation of the corresponding frequency matrices (b).** These matrices are extracted from chaos game representation of the *C. elegans* chromosome I.

The CGR technique reveals several hidden patterns that arise from distinct *k*-tuple compositions in DNA sequences. The frequency of occurrence of these patterns can be estimated by the use of the frequency chaos game representation (FCGR) [22]. The latter approach consists on dividing the CGR image into 4^*k*^ small squares where each sub-square is associated to a sub-pattern and has a side of 1/ 2^*k*^. The number of points in each sub-square thus created is then counted. This procedure allows extraction of the frequency of *k*-length words occurrence by dividing the number of dots onto the correspondent sub-squares by the complete length of the DNA sequence. To visualize the frequencies of occurrence of associated patterns, a normalized colour scheme is used. The darker pixels in the FCGR images represent the most frequently used words; otherwise, the clearest ones represent the most avoided words [23]. The Figure 2 is divided into two blocks where the first block illustrates the arrangement of oligomers in the FCGR’s sub-squares for *k*= {1, 2, 3}, and the second one is related to the frequency chaos game representations calculated for the chromosome I of the organism *C. elegans*.

Although representations based on the chaos game theory (we mean CGR and FCGR) have been successfully applied to a wide range of problems, their capacity in following the evolution of frequencies along DNA sequences remains, so far, totally unexplored. This motivates us to exploit the FCGR method in building signals in such a way that we can follow the frequency evolution of oligomers through a given sequence. We give a particular name to these signals—the FCGSs. This new mapping technique is based on assigning the frequency of occurrence of each oligomer to the same sub-pattern that exists in the sequence. For this purpose, two steps are required:

• The first step consists in the generation of the *k* th-order FCGR for the entire sequence. The FCGR matrix is expressed as follows:

2 $${\text{FCGR}}_{k}={\left[f_{i,j}\right]}_{1\leq i\leq }2^{k},{}_{1\leq j\leq }2^{k}$$

where *f*_*i*,*j*_ is the frequency value of the word situated at the intersection of the *i* th row and the *j* th column in the *k*-mer matrix.

• The second step consists in reading the input sequence by a group of successive *k*-nucleotides and replacing them by the corresponding frequency already calculated in the FCGR_*k*_ matrix.

In this sense, an FCGS_*k*_ can be generated by

3 $${\text{FCGS}}_{k}[\;n,i,j]=\sum_{n=1}^{L}{\text{FCGR}}_{k,i,j}[\;n].U_{{\text{motif}}_{k,i,j}}[\;n]$$

Here, *k* is the frequency chaos game representation’s order and FCGR_*k*,*i*,*j*_ refers to the FCGR_*k*_’s element which is placed at the intersection of the *i* th row and the *j* th column. Regarding an illustrative example of the FCGS technique, we consider the sequence *S*= {TTTTAGT GAAGCTTCTAGAT}. To encode *S* by FCGS _1_, FCGS _2_ and FCGS _3_, we must calculate the FCGRs matrices for orders 1, 2 and 3. Then, we extract all the oligomers of length {1, 2 and 3}, and we attribute for each of the monomers, dimers and trimers its occurrence frequency from the convenient frequency matrix. In this case, we enumerate 20 monomers, 19 dimers and 18 trimers. For illustration, we only consider 18 oligomers which are:

• Monomers = {T, T, T, T, A, G, T, G, A, A, G, C, T, T, C, T, A and G}

• Dimers = {TT, TT, TT, TA, AG, GT, TG, GA, AA, AG, GC, CT, TT, TC, CT, TA, AG and GA}

• Trimers = {TTT, TTT, TTA, TAG, AGT, GTG, TGA, GAA, AAG, AGC, GCT, CTT, TTC, TCT, CTA, TAG, AGA and GAT}

The associated frequencies are:

• Monomer frequencies = {0.45,0.45,0.45,0.45,0.25,0.2, 0.45,0.2,0.25,0.25,0.2,0.1, 0.45,0.45,0.1,0.45,0.25,0.2}

• Dimer frequencies = {0.2632,0.2632,0.2632,0.1579, 0.2105,0.1053,0.1053,0.1579, 0.1053,0.2105,0.1053,0.1579, 0.2632,0.1053,0.1579,0.1579, 0.2105,0.1579}

• Trimer frequencies = {0.1667,0.1667,0.1111,0.1667, 0.1111,0.1111,0.1111,0.1111, 0.1111,0.1111,0.1111,0.1111, 0.1111,0.1111,0.1111,0.1667, 0.1111,0.1111}.

**Figure 3:**
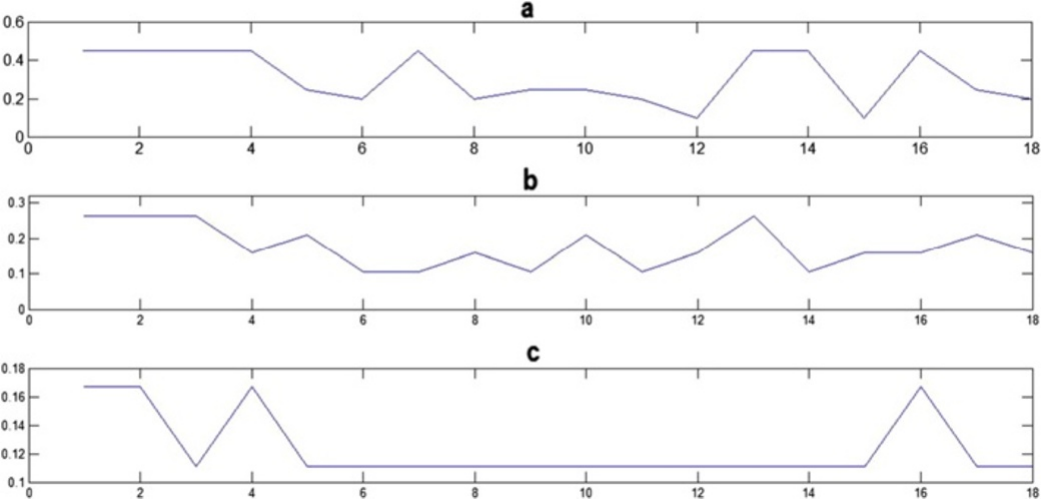
***FCGS***_***1***_ **(a),** ***FCGS***_***2***_ **(b) and** ***FCGS***_***3***_ **(c) corresponding to the sequence {TTTTAGTGAAGCTTCTAGAT}.**

At the end, we obtain three different signals, which are illustrated in Figure 3.

Note that increasing the FCGS order induces a more smoothed signal which is useful in capturing the important underlying patterns [24]. The smoothing is often used in enhancing the long-term trends that can be hidden in the original signal. This makes our coding technique suitable for fine studies. To demonstrate the effectiveness and usefulness of our coding, we chose to apply the complex Morlet wavelet analysis. By such application, we will note the smoothing effect in determining the characteristic patterns of certain areas of the DNA.

## 3 The wavelet transform analysis

The wavelet transform (WT) was introduced by Morlet in 1983 to study seismic signals. Then, the proposed processing was well formalized in 1984 with contributions of Grossman [25]. Therefore, the wavelet theory has been the subject of diverse theoretical developments and practical applications. In this section, we focus on the application of wavelet transform on the *C. elegans* genome aiming to explore its composition.

### 3.1 3.1 The continuous wavelet transform

The CWT of an arbitrary signal is a linear operation that consists in projecting the signal *x*(*t*) onto a wavelet basis. Mathematically, the CWT is given by Equation 4:

4 $$\;\begin{array}{c} W_{a,b}[\;x(t)]=\frac{1}{\sqrt{a}}\overset{+\infty }{\underset{-\infty }{\int }}x(t)\psi^{*}\left(\frac{t-b}{a}\right)\text{dt},a\in {\mathbb{R}}^{*+},b\in \mathbb{R} \end{array}$$

where *a* (*a*>0) and *b*(b∈ℝ) are respectively the scale and the time-shift parameters. Here, $\psi \left(\frac{t-b}{a}\right)$ is a scaled and shifted version of the so-called mother wavelet function *ψ*(*t*). Mother wavelet *ψ*(*t*), which is a wave-like oscillation, can be extended to its daughter wavelets in terms of the shift parameter *b* and the scale parameter *a*:

5 $$\psi_{a,b}(t)=\frac{1}{\sqrt{a}}\psi \left(\frac{t-b}{a}\right)$$

At fixed-scale and translation parameters (*a* and *b*), the wavelet transform coefficient, denoted by *W*_(*a*,*b*)_, represents the inner product of the daughter wavelet and the signal; this operation measures the degree of their resemblance at the concerned point. If *x*(*t*) is equal to *ψ*_(*a*,*b*)_(*t*), the wavelet coefficient is set to 1. Hence, the closer to 1 the coefficient is, the stronger the similarity will be.

Mother wavelets are band-pass filters that oscillate in the time domain it expands or compresses depending on the scale value. When *a* is large, the mother wavelet becomes stretched and serves for the high frequencies’ detection. In this case, the resolution of the time domain is low. On the contrary, when *a* is small, the mother wavelet is compressed, i.e. the frequency domain’s resolution becomes low in favor of the time domain’s resolution. Mathematically, the dilated and normalized mother wavelet function $\frac{1}{\sqrt{a}}$$\psi \left(\frac{t}{a}\right)$ will admit $\sqrt{a}\hat{\psi }(\mathrm{a\omega})$ as a Fourier transform, which explains the fact that an expansion in time induces a contraction in the frequency domain and conversely. This property makes analysis with wavelets a relevant tool for characterization of signals as well as for detection and identification of special spectral features. Mother wavelet function can be real or complex like in the case of complex Morlet wavelet which will be briefly described in the following.

### 3.2 3.2 The complex Morlet wavelet

The effectiveness of the wavelet transform in analyzing signals with complex nature (like in the case of genomic signals) depends on the choice of the basis function. In this study, our choice went to the complex Morlet wavelet. The advantage of the proposed mother wavelet is that it admits a parametrized bandwidth. This provides extra flexibility which ensures a good time-frequency resolution. The complex Morlet wavelet is a plane wave modulated by a Gaussian envelope and presents a quick attenuation [26] whose mother wavelet function is expressed as

6 $$\psi (t)=\pi^{-\frac{1}{4}}\left(e^{i\omega_{0}t}-e^{-\frac{1}{2}\omega_{0}^{2}}\right)e^{-\frac{1}{2}t^{2}}\;$$

where *ω*_0_ corresponds to the number of oscillations of the wavelet. Strictly speaking, *ω*_0_ must be greater than 5 to satisfy the admissibility criterion. This admissibility condition is required by all mother wavelets for the continuous wavelet transform to be invertible. Admissibility condition implies that the Fourier transform of the mother wavelet is 0 at frequency 0 [27]. This ensures the mother wavelet oscillates, which means that it acts as a band- pass filter. The Fourier transform of the complex Morlet wavelet function is given by

7 $$\hat{\psi }(\omega )=\sqrt{2}\pi^{\frac{1}{4}}e^{-\frac{1}{2}{\left(\omega -\omega_{0}\right)}^{2}}$$

At a fixed scale *a*, the complex Morlet wavelet and its Fourier transform are given by

8 $$\psi_{a,b}(t)=\frac{1}{a}\pi^{\frac{-1}{4}}\left(e^{-i\omega_{0}\frac{t-b}{a}}e^{\frac{1}{2}{\left(\frac{t-b}{a}\right)}^{2}}\right)$$

9 $${\hat{\psi }}_{a,b}(\omega )=\sqrt{2}\pi^{\frac{1}{4}}e^{-\frac{1}{2}{\left(\mathrm{a\omega}-\omega_{0}\right)}^{2}}$$

In the frequency domain, the wavelet coefficient is a wavelet filter characterized by the constant QFactor [28]:

10 $$\text{QFactor}=\frac{\mathrm{Center}\;\text{frequency}}{\text{Bandwidth}}$$

The central frequency of the mother wavelet, denoted by *f*_*c*_, is the position of the global maximum of $\hat{\psi }(\omega )$ which is given by $f_{c}=\frac{\omega_{0}}{2\Pi }$. As for the bandwidth, denoted by *f*_*b*_, it is centered around *f*_*c*_ and controls the wavelet window [29]. The complex Morlet wavelet can be expressed by the following equation:

11 $$\psi (t)=\frac{1}{\sqrt{\pi f_{b}}}e^{i2\pi f_{c}t}e^{-\frac{t^{2}}{f_{b}}}$$

To allow easy graphical interpretation, it is preferred to display the modulus of the CWT coefficients: |*W*_(*a*,*b*)_|. This representation is called a scalogram and it represents the amplitude information of the signal at each scale *a* and position *b*. The scalogram can also be depicted in the time-frequency domain instead of the time-scale domain by converting the scales to frequencies using the formula:

12 $$f_{c}=\frac{\omega_{0}}{2\mathrm{\pi a}}$$

Thus, a scalogram is a 2D plot where time is on the horizontal axis, frequency on the vertical axis, and amplitude of CWT coefficients are colored according to a defined code. In the following section of this paper, we will focus on analyzing the Morlet scalogram.

## 4 Results and discussion

In this work, we focus our study on the analysis of DNA sequences within the *C. elegans* genome. The genomic sequences are extracted from the NCBI database [30]. As for the mapping technique, we choose the FCGS algorithm with the three first levels. Thus, the generated signals are FCGS_1_, FCGS_2_ and FCGS_3_ of the whole chromosomes. Concerning the wavelet analysis, we use the complex Morlet wavelet with a support size of 1,420. Application of the continuous wavelet transform on the appropriate sequences is accomplished along 64 scales by using a mother wavelet centered on *ω*_0_=5.4285 (radian units).

**Figure 4:**
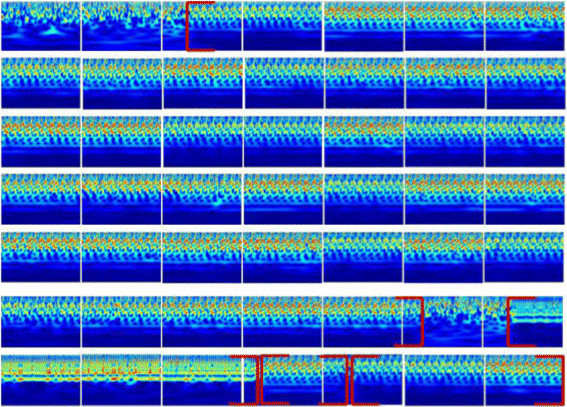
**The scalogram representations of a sequence on the chromosome III of** ***C. elegans*** **.** Coded by FCGS_2_ (position [7403001–7452000]).

Close inspection of the resulting scalograms shows the role played by this analysis in the characterization of different sites along the DNA sequences. In fact, we offer a standard way to represent genomes and reveal the biological hotspots, regardless of their nature or their length. Through a simple zooming of 10^3^ bp, we are able to observe different features with great precision. Even the finer details are easily discerned. Several regions are visually distinguished by typical motifs which include prominent periodicities. We analyze these regions in the NCBI database [30] to ascertain their nature. Besides, it is important to note that not all revealed stretches are identified; there are some regions that we have not succeeded in understanding the related biological significance. For example, in Figure 4, we provide a series of scalograms which represent a sequence taken from the chromosome III of *C. elegans*. As we can see, this example well illustrates the presence of different DNA structures which are easily observed due to their specific behaviors (the red brackets delimit the boundaries of these elements). According to the NCBI database, the prominent signatures relate to the elements CeRep59 (37,899 bp), CeRep55 (3,797 bp), CeRep59 (1,091 bp) and CeRep59 (2,844 bp).

Among the structures that possess particular signatures, we selected some elements of the *C. elegans* chromosome I to study them, namely: intron, STS and Cerp3 elements.

### 4.1 4.1 Intron signature

It is well-known that the genomic sequences present a strong three-base periodicity. The latter periodicity is an interesting feature of the protein-coding regions (exons). Several signal processing approaches and computational algorithms have been developed based on this periodicity for predicting exons. Most of the coding region prediction methods used the discrete Fourier transform (DFT)-based algorithms through which exons refer to the maximum of the Fourier power spectrum at the position of 1/3 frequency [31]-[35]. In the same context, performing the DFT on the wavelet coefficient of the correlation function at frequency 1/3 has improved the peaks that mark exons in the Fourier spectrum [36]. On the other hand, for identification of protein coding regions, the use of the CWT based on the modified Morlet wavelet has provided more accurate results [7],[37]. All of these works revolve around exon prediction; whereas intron prediction has not yet drawn the attention it deserves (the intron is a non-coding region in eukaryotic gene).

**Figure 5:**
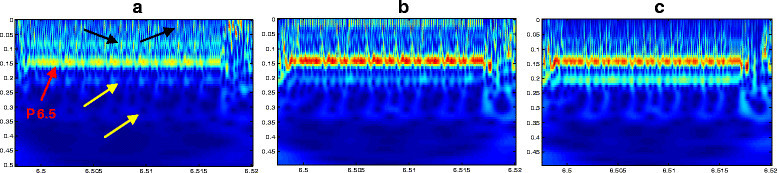
**Scalograms of an intron found in the** ***C. elegans*** **gene Y65B4A.2.** Coded with FCG*S*_1_**(a)**, FCG*S*_2_**(b)** and FCG*S*_3_**(c)**.

The novelty in our work consists in providing an efficient way to represent main characteristics of intronic sequences. Indeed, the FCGS coding highlights motifs having different forms with a high level of energy around specific frequency values. In our work, we found that most of introns in the *C. elegans* genome present high energy around the frequency 1/6.5. Figure 5 presents an illustrative example of an intron found in the *C. elegans* chromosome I (position [649752–652010]).

This example (Figure 5a,b,c) exposes the behavior of a typical intron which is characterized by the presence of specific motifs with high energy around the frequency 1/6.5 (as shown by the red arrow; P denotes periodicity) [38],[39]. Other periodic motifs are also apparent at the level of harmonics which are marked by a lower intensity line. We note that the intensity of the lower harmonics (as indicated by the yellow arrows) increases by increasing the order of the FCGS coding. Otherwise, the intensity of the upper harmonics (see the black arrows) decreases by increasing the order of the FCGS coding. From this example, we can see that this intron presents a remarkable behavior within the three levels of FCGS despite the smoothing effect of higher order FCGSs (especially noted when we code with FCGS_3_).

### 4.2 4.2 STS signature

**Figure 6:**
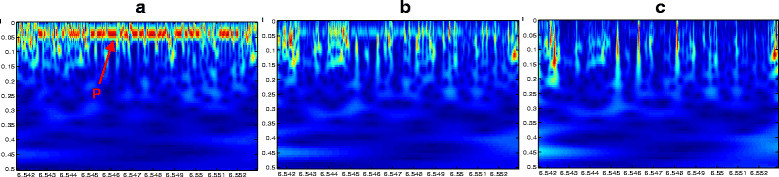
**Scalograms of a sequence-tagged site (STS).** Coded by FCG*S*_1_**(a)**, FCG*S*_2_**(b)** and FCG*S*_3_**(c)**.

Traditional gene mapping techniques are slow and painstaking. The discovery of the sequence-tagged sites (STS) have opened a new way for geneticists to speed up the establishment of genetic and physical mapping of genes along chromosomes. An STS is a specific region of DNA which can be uniquely identified through its sequence. In addition, it is an easily PCR-amplified sequence which can contain repetitive elements as microsatellites. For the analysis of this abundant class of DNA, we choose the example of Figure 6.

By examining the FCGS_1_ result (Figure 6a), we can note the presence of periodic patterns with high energy at the top of the scalogram (which is indicated by the red arrow). These patterns are located within a considerable frequency band. If we consider the FCGS_2_ result, we can see that the energy level of the frequency band is weakened (Figure 6b). This is due to the smoothing property of the FCGS coding. The smoothing effect of the FCGS_3_ is also noticed in Figure 6c.

### 4.3 4.3 Cerp3 signature

**Figure 7:**
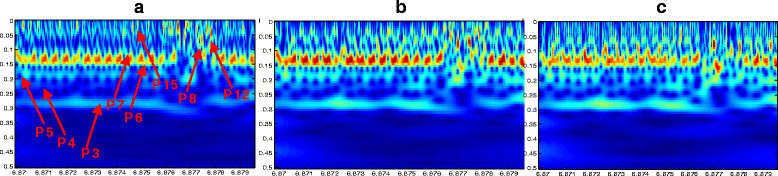
**Visualization of the repetitive element Cerp3 by complex Morlet scalogram.** FCG*S*_1_**(a)**, FCG*S*_2_**(b)** and FCG*S*_3_**(c)** codings.

The last example that we are studying here is part of the Cerp3 repetitive family. The Cerp3 DNA consists of dispersed repeated elements with a length of about 1,000 bp and presents 50 to 100 copies in the *C. elegans* genome. Such a nematode segment hides specific periodicities that we are disclosing in the related scalograms (Figure 7).

All the scalograms, strikingly, display a long chain of motifs consisting of seven- and six-base periodicities. Figure 7a (related to the FCGS_1_ coding) shows other patterns including strong periodicities on the top of the scalograms. As for the FCGS_2_ coding (Figure 7b), it enhances periodicities of 5 bp and 3 bp and shows up other periodicities corresponding to the 15-, 12- and six-base repetitive elements. Finally, Figure 7c underlines the contribution of the FCGS_3_ scheme in the enhancement of periodicities like 15, five and four bases.

## 5 FCGS and the local signatures in *C. elegans*

In this work, we have investigated the important role of color scalograms which offer an easy visual navigation through genomic sequences. Thus, we have exposed the behavior adopted by some DNA sequences in the time-frequency plan which turns out to be easily characterized by the presence of different periodic patterns within the FCGSs scalograms. These behaviors appear as strong local signatures within the genome. As we have seen, there are some signatures which strongly appear only when we code with FCGS_1_ and other signatures that similarly appear within the three levels of FCGSs.

Aiming at studying the role of the FCGS order in the enhancement of the DNA signature, we consider the contribution of the percentage of the frequency band which specifies the DNA signature in terms of energy measure. This choice went to the fact that the energy of the characteristic sub-band is one of the main statistical features that can be extracted from the wavelet domain as texture descriptor [40]. The study is performed with three examples of each of the intron, STS and Cerp3 sequences (see Table 1). These sequences are coded by the frequency chaos game signal order 1, 2 and 3.

**Table 1:** **Position and frequency band of the introns, STS and Cerp3 sequences in the** ***C. elegans*** **chromosome I**

	Position of the sequences in the***C. elegans***chromosome I			
Structures	Sequence 1	Sequence 2	Sequence 3	Frequency band
Introns	649752–652010	669573–671806	692688–693513	0–0.33
STS	3651199–3652332	3654158–3655291	7385764–7386961	0–0.2
Cerp3	953661–954106	593817-594993	686985–687959	0–0.28

To be able to evaluate the energy contribution of the different periodic patterns in these sequences, we have to fix the frequency band limit in such a way that it includes all the periodic motifs (see Table 1).

**Figure 8:**
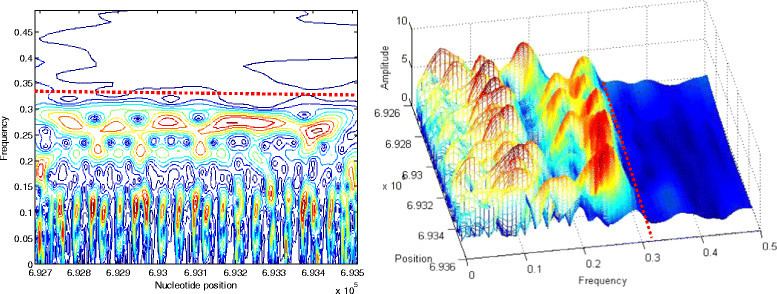
**Contour and 3D visualizations of prominent frequency band of intron 3 when coded by** ***FCGS***_***2***_ **.**

**Figure 9:**
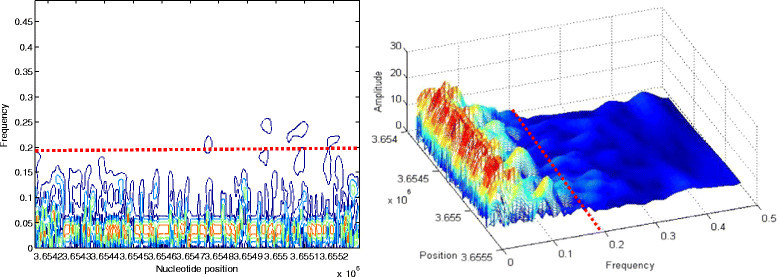
**Contour and 3D visualizations of prominent frequency band of STS 2 coded by** ***FCGS***_***1***_ **.**

**Figure 10:**
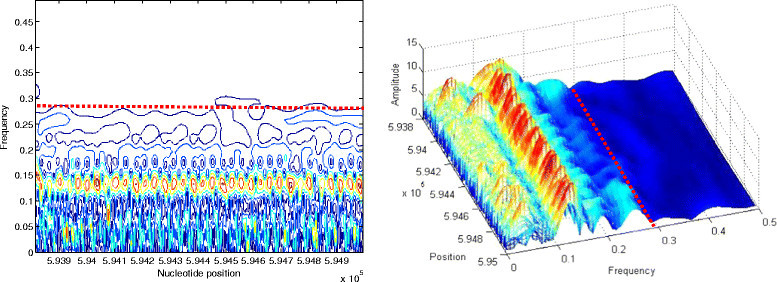
**Contour and 3D visualizations of prominent frequency band of second Cerp3 coded by** ***FCGS***_***2***_ **.**

The choice of the frequency boundaries is justified by the contour and the 3D plots given in Figures 8, 9 and 10. The dashed red lines in these figures delimit the characteristic frequency band. Figure 8 refers to the third intron when it is coded by FCGS_2_.

In Figure 9, we provide the pattern distribution of the STS 2 sequence (coded by FCGS_1_) through the contour and the 3D plots.

Finally, Figure 10 shows the contour and the 3D plots of the second Cerp3 sequence (coded by FCGS_2_).

The second part of this study consists in the measurement of the strongest motifs’ energy distribution for the intron, STS and Cerp3 sequences coded by the frequency chaos game signals order 1, 2 and 3. Thus, we calculate the total energy of the scalogram (which is designated by *E*_*t*_) and the energy measure of the prominent frequency sub-band (which is designated by *E*_*p*_). The contribution of this sub-band energy is then weighted by the percentage ratio between them.

**Figure 11:**
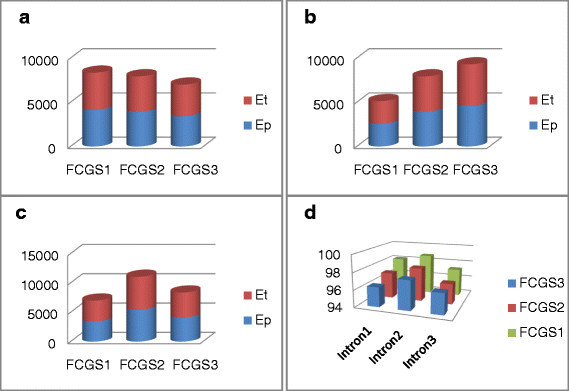
**Characteristic energy contribution of three introns when coded with** ***FCGS***_***1***_**,** ***FCGS***_***2***_**and** ***FCGS***_***3***_**.** **(a)** Intron 1, **(b)** intron 2, **(c)** intron 3, **(d)** ratio.

In Figure 11, we provide the energy’s values, which are calculated over a portion of 800 bp for the three introns. Based on the histogram plots, we deduce that the partial energy is so close to the total energy for all introns. In addition, FCGS_1_, FCGS_2_ and FCGS_3_ yield close percentage values, which confirm the fact that they similarly characterize introns.

**Figure 12:**
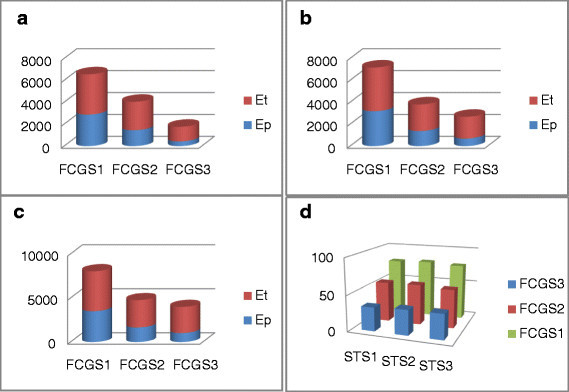
**Complex Morlet scalograms of three sequence-tagged sites (STS) coded by** ***FCGS***_***1***_**,** ***FCGS***_***2***_**and** ***FCGS***_***3***_**.** **(a)** STS 1, **(b)** STS 2, **(c)** STS 3, **(d)** ratio.

As for the STS sequences, the scalograms show that the FCGS_1_ is better suited to study this DNA type. To prove this, we consider the contribution of the characteristic patterns relating to the three first levels of FCGS. In terms of energy percentage, we provide the contribution of the characteristic patterns relating to the FCGS scalograms in Figure 12. The energy values are calculated over a portion of 1,134 bp.

Note that the energy values considerably decline when the FCGS order increases for all the STS sequences. The ratio values prove, in addition, that FCGS_1_ is the only coding that characterizes STS sequences.

**Figure 13:**
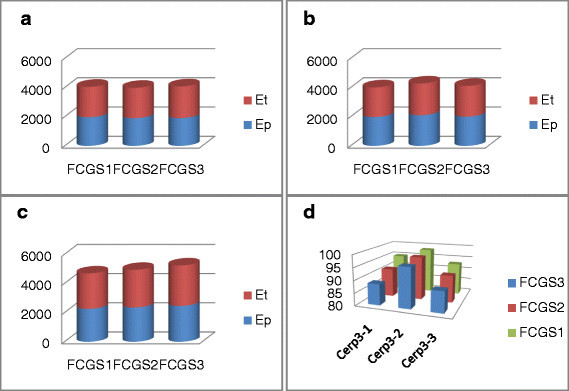
**Characteristic energy contribution of the considered Cerp3 sequences.** **(a)** Cerp3-1, **(b)** Cerp3-2, **(c)** Cerp3-3, **(d)** ratio.

Finally, the energy values of the Cerp3 sequences (through a portion of 445 bp) are provided in Figure 13. From the latter histograms, we can deduce that the FCGS order 1, 2 and 3 allow the Cerp3 characterization, which results in close energy values.

Aside the qualification of these sequences by a specific signature, there are many DNA classes that are easily distinguished by relevant motifs in the scalograms. Therefore, based on the study of significant homology between signatures, we can establish efficient algorithms for DNA recognition and classification.

## 6 Conclusion

DNA coding methods play a major role in revealing information about significant biological sequences. However, the choice of such methods depends on the features that they can reflect. It appears that the available mapping techniques rely mostly on the 3-bp or 10-bp behaviors and are not well adapted to examine all periodic structures contained in the complex nature of DNA. In this context, we introduced a new mapping technique, aiming to characterize a wealth of DNA sequences. The proposed method is based on the chaos game theory and we refer to it as FCGS. The FCGS coding consists in assigning the frequency of occurrence of each sub-pattern to the same group of nucleotides that exist in the DNA sequence. Such a mapping has the advantage of providing a multitude of signals which offer the possibility to treat the DNA sequence from different views, taking into account the statistical properties of resident oligomers.

The performance of the FCGS scheme in terms of information revelation from DNA sequences was tested by the continuous wavelet transform. The complex Morlet wavelet was employed to create color scalograms for the *C. elegans’* FCGSs (order 1 to 3).

By reviewing the resulting scalograms, we found that the selected wavelet transform readily identifies different DNA structures. Several hidden periodicities and features which cannot be revealed by classical DNA analysis methods (such as the STFT) were sharply identified. Simulation results show a pronounced 6.5 base period in intergenic residues, more specifically in intronic ones. However, there are other introns which include periodicities like 5 bp and 3 bp. These periodicities are derived from a specific organization of periodic patterns forming thus a local signature. Through this study, it is shown that the variable patterns observed in the intron DNA are all exhibited by the FCGS_1_, FCGS_2_ and FCGS_3_ codings. Besides introns, we have shed the light on another type of DNA sequences: the STS. The STS are particular DNA sequences recently used in the gene mapping procedures. When we code with an FCGS order 1, we managed to find a special signature of this DNA class that derives from the microsatellite repetitive elements that it contains.

Overall, in the mapping efforts for the nematode *C. elegans*, various classes of repetitive DNA were annotated. Among them, we considered a particular class of *C. elegans* dispersed repeats: the Cerp3. The related scalograms provide clear periodical motifs of seven- and eight-base repeats. This time-frequency signature is illustrated when the coding schemes FCGS_1_, FCGS_2_ and FCGS_3_ are used.

In conclusion, the results stemming from the complex Morlet wavelet analysis of the FCGSs have showed its accuracy in detection of variable DNA structures. Moreover, this could serve in discovering unknown domains with potential biological significance in genomes.

## Authors’ original submitted files for images

Below are the links to the authors’ original submitted files for images. Authors’ original file for figure 1 Authors’ original file for figure 2 Authors’ original file for figure 3 Authors’ original file for figure 4 Authors’ original file for figure 5 Authors’ original file for figure 6 Authors’ original file for figure 7 Authors’ original file for figure 8 Authors’ original file for figure 9 Authors’ original file for figure 10 Authors’ original file for figure 11 Authors’ original file for figure 12 Authors’ original file for figure 13 Authors’ original file for figure 14 Authors’ original file for figure 15 Authors’ original file for figure 16 Authors’ original file for figure 17 Authors’ original file for figure 18 Authors’ original file for figure 19
