# Supplementary figures and images for: Wavelet analysis of frequency chaos game signal: a time-frequency signature of the C. elegans DNA
Source: EURASIP J Bioinform Syst Biol. 2014 Sep 12;2014:16. doi: 10.1186/s13637-014-0016-z (PMC5270495; doi:10.1186/s13637-014-0016-z)

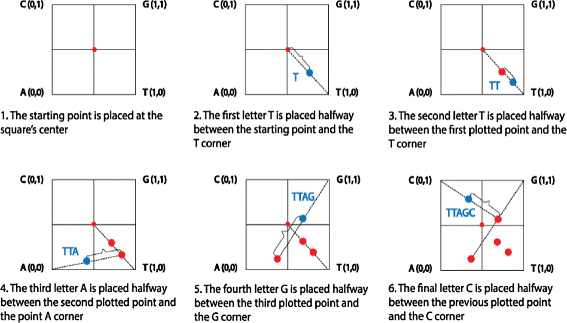

Supplement: Supplementary file 1 — Authors’ original file for figure 1 [file 13637_2014_16_MOESM1_ESM.gif]

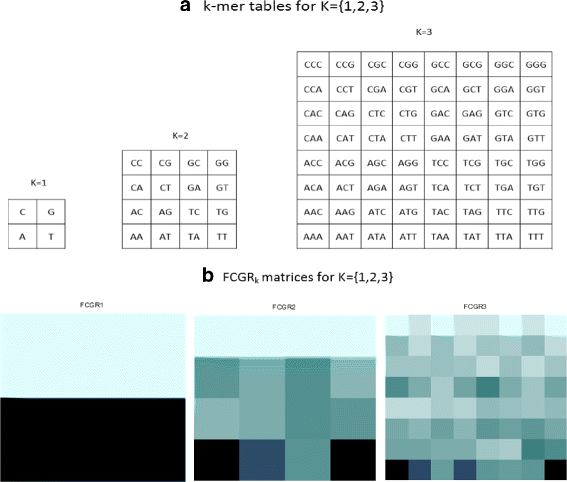

Supplement: Supplementary file 2 — Authors’ original file for figure 2 [file 13637_2014_16_MOESM2_ESM.gif]

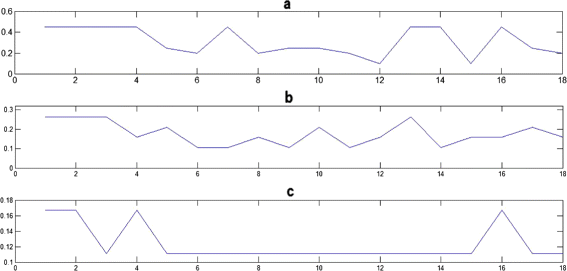

Supplement: Supplementary file 3 — Authors’ original file for figure 3 [file 13637_2014_16_MOESM3_ESM.gif]

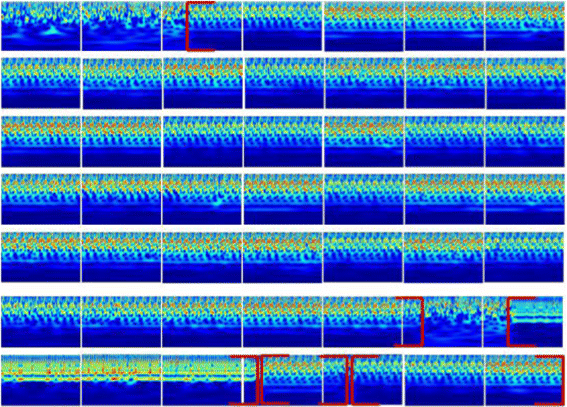

Supplement: Supplementary file 4 — Authors’ original file for figure 4 [file 13637_2014_16_MOESM4_ESM.gif]

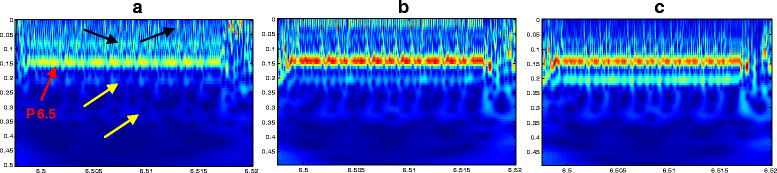

Supplement: Supplementary file 5 — Authors’ original file for figure 5 [file 13637_2014_16_MOESM5_ESM.gif]

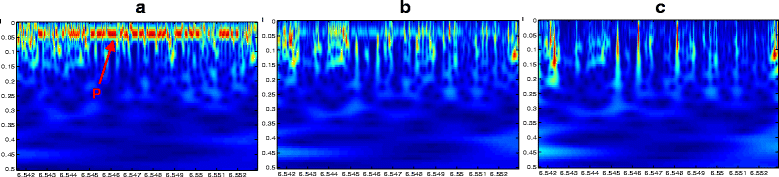

Supplement: Supplementary file 6 — Authors’ original file for figure 6 [file 13637_2014_16_MOESM6_ESM.gif]

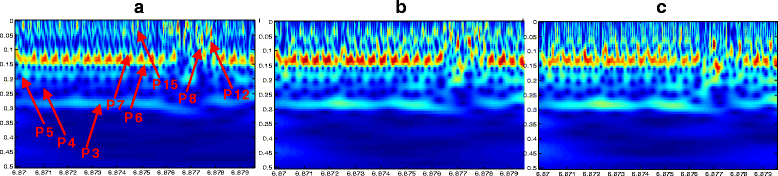

Supplement: Supplementary file 7 — Authors’ original file for figure 7 [file 13637_2014_16_MOESM7_ESM.gif]

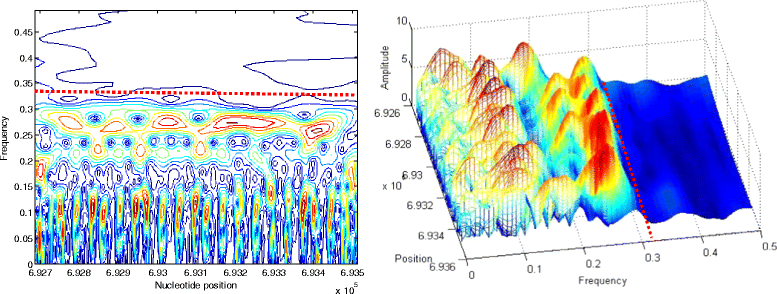

Supplement: Supplementary file 8 — Authors’ original file for figure 8 [file 13637_2014_16_MOESM8_ESM.gif]

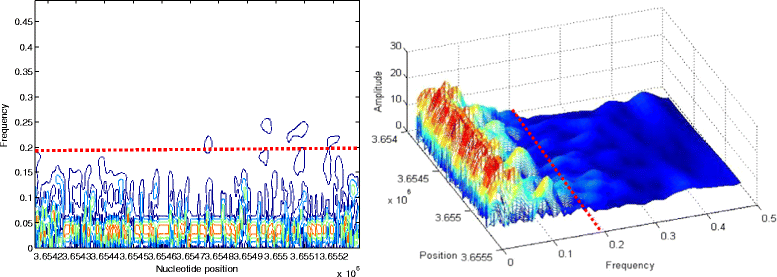

Supplement: Supplementary file 9 — Authors’ original file for figure 9 [file 13637_2014_16_MOESM9_ESM.gif]

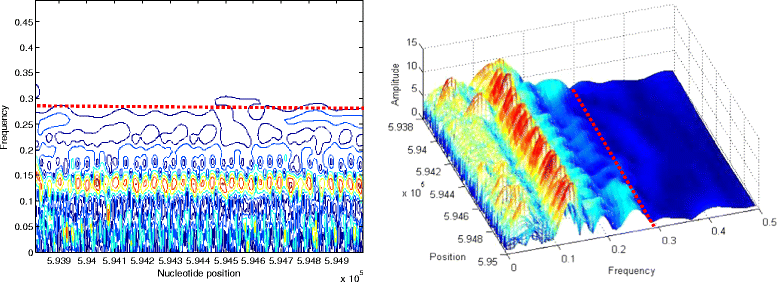

Supplement: Supplementary file 10 — Authors’ original file for figure 10 [file 13637_2014_16_MOESM10_ESM.gif]

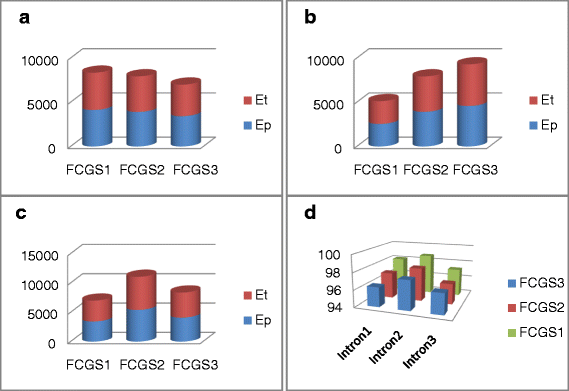

Supplement: Supplementary file 11 — Authors’ original file for figure 11 [file 13637_2014_16_MOESM11_ESM.gif]

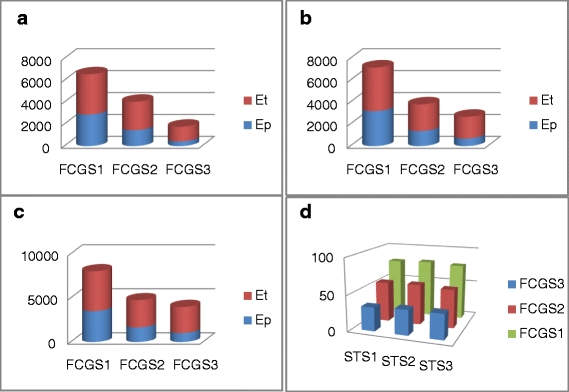

Supplement: Supplementary file 12 — Authors’ original file for figure 12 [file 13637_2014_16_MOESM12_ESM.gif]

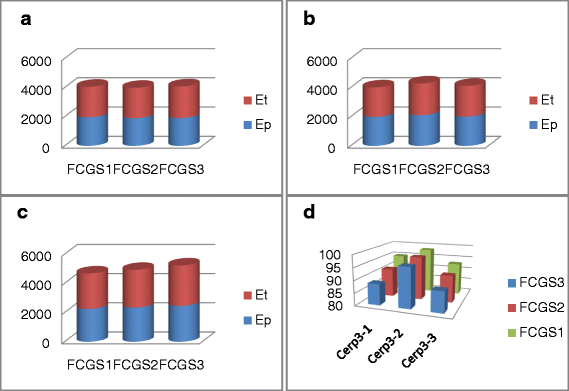

Supplement: Supplementary file 13 — Authors’ original file for figure 13 [file 13637_2014_16_MOESM13_ESM.gif]

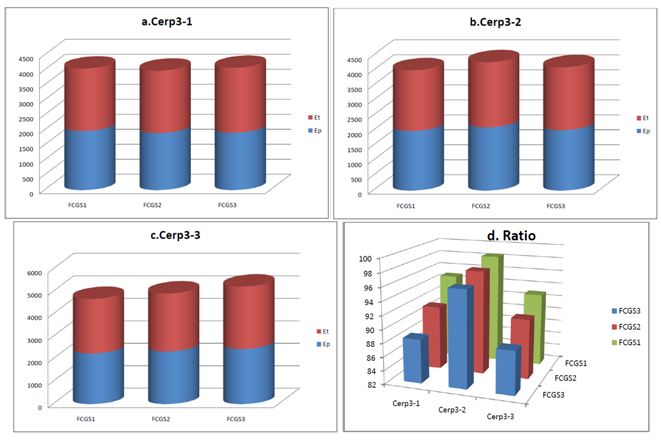

Supplement: Supplementary file 14 — Authors’ original file for figure 14 [file 13637_2014_16_MOESM14_ESM.jpeg]
